# Interannual site fidelity by Svalbard walruses

**DOI:** 10.1038/s41598-024-66370-w

**Published:** 2024-07-09

**Authors:** Lonnie Mikkelsen, Kit M. Kovacs, Marie-Anne Blanchet, Gary Brodin, Christian Lydersen

**Affiliations:** 1 Norwegian Polar Institute, Framsenteret, Hjalmar Johansens Gate 14, 9296 Tromsø, Norway; 2 Pathtrack Ltd, Unit 1, Chevin Mill, Leeds Road, Otley, LS21 1BT UK

**Keywords:** Animal migration, Behavioural ecology

## Abstract

The Arctic is experiencing rapid reductions in sea ice, affecting all ice-dependant species. In the present study we examine interannual seasonal movements and habitat use in relation to sea ice coverage for one of the Arctic endemic marine mammals. We tagged 40 male walruses (*Odobenus rosmarus*) in the Svalbard Archipelago with custom-designed tusk-mounted GPS loggers. Twelve of these animals provided tracks that lasted 1–6 years. Eleven of the walruses displayed clear seasonal migratory behaviour between summer foraging areas and winter breeding areas. Individuals showed high inter-individual variation, but clear site fidelity, using the same areas in consecutive years despite variable sea ice conditions. The walruses swam 5225–10,406 km per year and travelled remarkably similar distances between years on an individual basis. The phenology of migration was not impacted by sea ice concentrations or daylight length but was consistent at the individual level, suggesting endogenous drivers. Sea ice concentrations influenced movement behaviour with animals showing more tortuous paths when in areas with heavy sea ice, possibly searching for polynyas where females reside. Ongoing climate change is expected to drastically change walrus habitat, and it remains to be seen if walruses will be able to shift from their fixed seasonal migratory routines.

## Introduction

The Arctic is changing rapidly due to global warming with declines in sea ice being one of the most visible and notable results^1^. Given that all Arctic endemic marine mammals depend on this substrate for most or part of their life cycle, it is essential to study their behaviour to understand how the changes to their habitat are affecting populations. Walruses (*Odobenus rosmarus*) are endemic to Arctic and sub-Arctic regions. They are benthic foragers that feed mainly on bottom-dwelling invertebrates in relatively shallow waters^2,3^. Walruses occur as two distinct subspecies: the Pacific walrus (*O. r. divergens)* which occurs in the Arctic and sub-Arctic waters of the Chukchi, Bering and Laptev seas (USA and Russia)^4^ and the Atlantic walrus (*O. r. rosmarus*), which inhabits coastal areas in north-eastern Canada, Greenland, Svalbard (Norway), and the Barents and Kara Seas (western part of Arctic Russia)^5^. The walruses inhabiting Svalbard and Franz Josef Land (Russia) are a genetically distinct population^6^. This population was at the verge of extinction before it became protected from hunting in 1952^7^. A marked recovery has taken place since then and aerial surveys of the Svalbard fraction of the population have documented that numbers have increased from 2629 (CI: 2318–2998) in 2006^8^ to 5503 (CI: 5031–6036) in 2018^9^. The Svalbard fraction of the population is heavily dominated by males^10^, although the number of mother-calf pairs has been increasing recently in Svalbard, particularly in north-eastern areas within the archipelago^11^.

**Figure 1:**
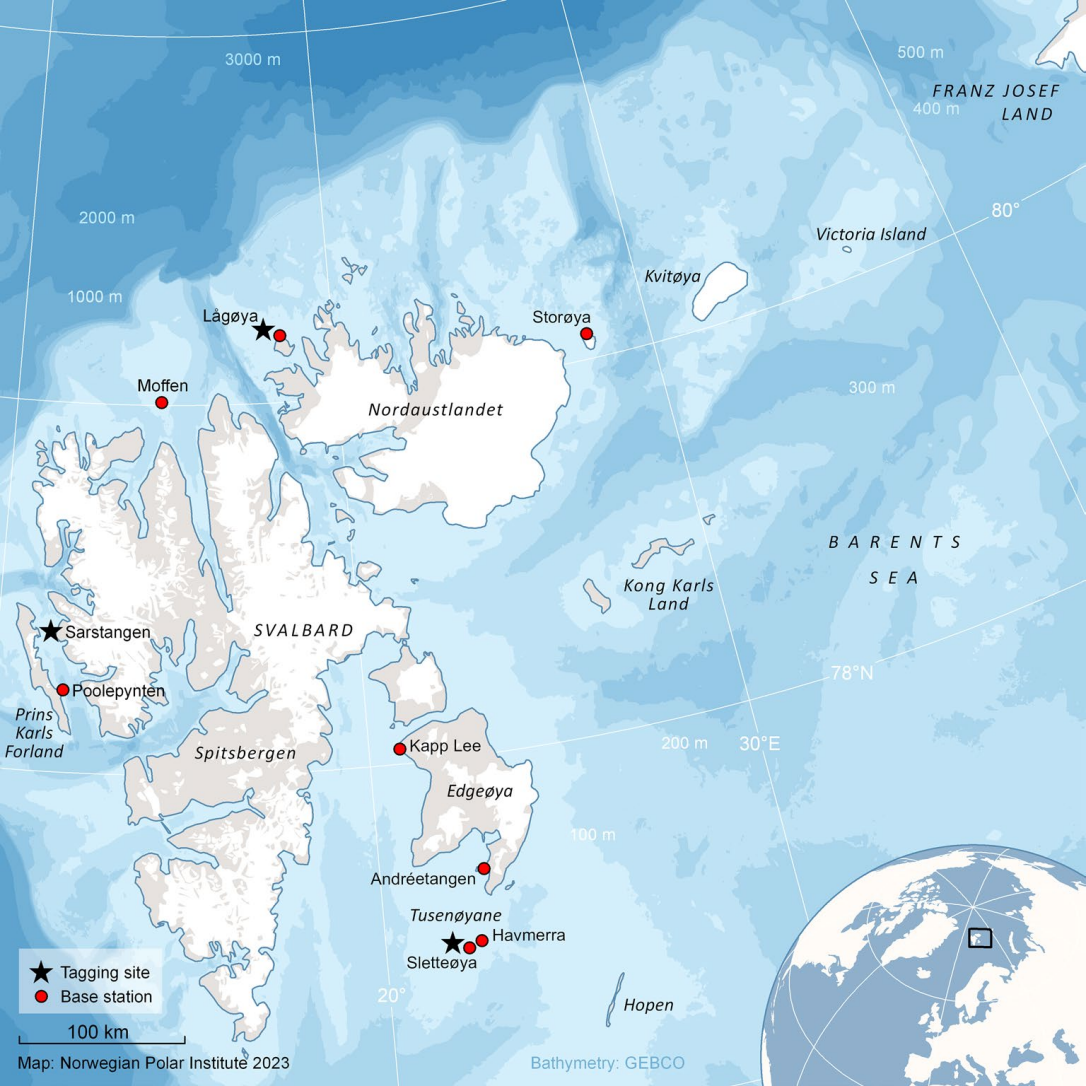
Map of Svalbard and eastern Franz Josef Land. Place names, tagging sites and locations of receiver stations are indicated. The map was generated in Esri ArcMap v10.8.1, https://desktop.arcgis.com/en/arcmap/index.html.

In the winter period, walruses undertake seasonal migrations to traditional mating areas^12–15^. While they generally follow the advance and retreat of sea ice formation^16^, they are also able to occupy areas deep into ice-covered waters^17–19^, although winter/breeding sites are often associated with less dense sea ice, such as polynyas^17^. Previous tracking studies conducted in Svalbard have demonstrated that male walruses migrate from various islands in the archipelago eastward to Kvitøya, Victoria Islands and Franz Josef Land (Fig. 1) in the autumn/winter^19,20^, to overlap with the female component of this population. Based on interpretation of tracks and diving information, several breeding areas deep into the ice between Svalbard and Franz Josef Land have been identified^15,19^.

Although the walrus population in Svalbard is increasing following protection from hunting, it is likely to be negatively impacted by the changes that diminishing sea ice is having on the ecosystem. Thus, information on current habitat use, and the range of individual behavioural plasticity, of walruses is essential. However, such information is currently very limited. A few long-term (> 1 year) tracking data sets from Svalbard indicate that animals return to more or less the same foraging grounds in the summer^19^. However, to what extent this is a general phenomenon and whether they return to same summering and wintering grounds over several years is not known.

Walruses are unique among pinnipeds in having tusks that afford a solid attachment site for biologging instruments^17,19–21^. However, the deployment of various biologgers on walruses has proven to be rather challenging, mainly due to problems with the chemical immobilisation and an associated high risk of mortality^22–25^. Recent improvements in chemical immobilization^26^ and biologging now allow for multi-year tracking of walruses, offering insight into stability of behaviour over time and how individuals may react to environmental drivers.

In this study we deployed custom-designed tusk-mounted GPS loggers that were designed to collect data over a five-year period, with the intention of studying seasonal movements and habitat use by walruses in relation to environmental drivers.

## Methods

### Tag specifications

**Figure 2:**
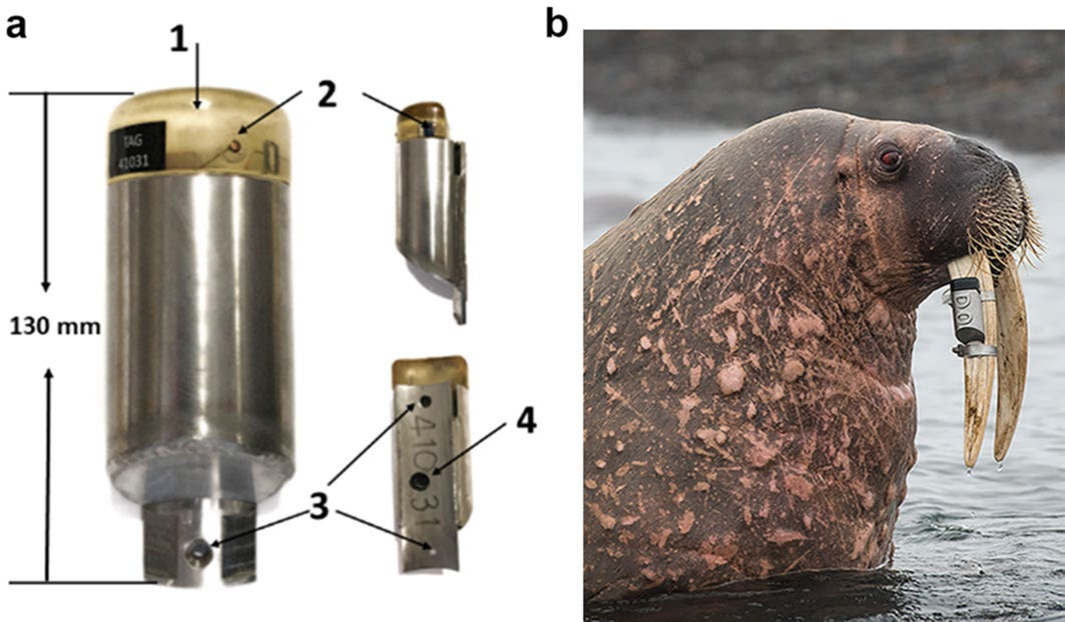
GPS tag and tag mounted on a walrus's tusk. Custom-designed Global Positioning System (GPS) tracking devices, developed in collaboration between the Norwegian Polar Institute, Tromsø, Norway, and Sirtrack (now Lotek.com) in Havelock North, New Zealand, were mounted on tusks of walruses in Svalbard, Norway. (**a**) front side and back view of the GPS logger. 1: Epoxy encapsulated GPS; 2: Saltwater switch; 3: holes for screws; 4: communication port. (**b**) Walrus with a tusk-mounted logger with ID D0. Picture taken 1-year post-deployment (photo by: Burney Iversen).

Custom-designed Global Positioning System (GPS) tracking devices, developed specifically for walruses in collaboration with Sirtrack (now Lotek.com) in Havelock North, New Zealand, were used to collect position data for walruses instrumented in Svalbard, Norway. The electronics, including the GPS and UHF antennas, were embedded in epoxy and encased in a stainless-steel housing to give the electronics maximum protection, and then the casings were flooded with polyurethane. The entire logger including an extension lip at the bottom is 130 mm long and weighs 0.65 kg (Fig. 2). The extension lip is part of a baseplate that protrudes up to the end of the stainless-steel casing. The lip has a hole in each end that accommodates a small screw that is set several mm into the tusk to prevent the logger from shifting position. At both ends of the baseplate a small rim prevents the hose clamps, that cover the heads of the screws and holds the tag to the tusk, from slipping off. Each tag had a unique ID number welded onto its surface (see Fig. 2b, ID D0) to enable individual identification if the animal carrying the tag was resighted.

The core software and hardware used in these tracking devices is the same as that used in Lotek Fastloc 3 products (https://www.lotek.com/products/fastgps-series/). The loggers collect GPS position data via a ceramic patch GPS antenna located at the head of the housing to obtain the best possible view of the GPS satellite constellation. An onboard saltwater sensor uses conductivity readings between the two probes shown in Fig. 2a to prevent the device from trying to acquire GPS signals when submerged. The devices were programmed to acquire a GPS fix every hour. Only when a dry reading is obtained from the saltwater sensor is a GPS acquisition attempted. If the sensor detects the tag is submerged it continually samples at a rate of 4 Hz until a dry reading is obtained. If the GPS acquisition (requiring a minimum of 4 satellites) is successful, a GPS position is logged; the tag also logs the time of attempted location fixes. The tags were fitted with primary Lithium batteries with an expected lifetime of 5 years on this data-sampling schedule.

GPS data collected by the loggers is downloaded wirelessly to receiver stations (mobile or stationary) via UHF at distances up to 500 m. Dynamic data download rates are used depending on the quality of the signal path between tag and base station; data transmission is being slower at longer distances. Each logger will download data only once to one of the receiver stations. If visiting another base station, only new data will be sent. The loggers were configured to search for a receiver station every 30 min. The tags first listen to determine if any other tags are already uploading data. If another UHF upload session is detected, the tag skips until the next time check. If no other UHF session is detected, the tag will transmit to see if it receives a response from a receiver station to initiate data upload, and if so, will start downloading data.

Stationary receiver stations were established at eight different haul-out sites for walruses in Svalbard (base stations, Fig. 1). In addition, portable receiver stations were carried by various field parties to enable on-site downloading from walruses observed carrying a GPS logger at sites without base stations. The receiver stations were equipped with large primary Lithium batteries (6 D-cells a 3.9 V- SAFT LHS-20) with an expected lifetime of at least 12 months, while the mobile receiver stations employed rechargeable Lithium Polymer batteries. The receiver stations’ batteries were replaced annually, at which time all new data on the stations were downloaded to a laptop. Both fixed and mobile receiver stations had the same core hardware, with each device being able to store approximately 250,000 GPS locations between downloads. Once the data from a receiver station were successfully downloaded to a laptop, the full memory capacity was once again available for future data downloads from tags. The tags had capacity to store up to 60,000 locations in onboard memory, which was beyond the expected lifetime of the tags.

### Tagging procedure

A total of 40 adult male walruses were instrumented with GPS loggers during 19–25 August 2014 at Sletteøya (N = 20), 3–4 August 2015 at Sarstangen (N = 4) and 8–12 August 2015 at Lågøya (N = 16). All of these sites are sandy or rocky beaches where walruses routinely haulout in summer (see Fig. 1). Animals at the landside edge of a group were selected for tagging to prevent the animal having open access to the water if frightened by the injection dart. Animals were also chosen based on having large tusks to carry the logger and they had to be on flat ground in an area clear of obstacles (rock or logs) that might prevent us from rolling the darted animal onto its belly for easy access to the epidural vein for injection of the reversal agent. All walruses were darted with a CO_2_ propelled Teledart RD 706 injection gun^27^ in the upper thigh, or lower back muscles from a distance of 5–10 m. All animals received a standard dose of 7.8 mg etorphine hydrochloride regardless of body size^26^. Once a walrus was immobilized, the GPS logger was mounted to a tusk using HI-TORQUE™ heavy-duty stainless steel hose clamps (JCS Hi-Torque Ltd., Suffolk, England) and Sicaflex epoxy. Two small holes were drilled in the outer enamel of the tusk. A screw with a smaller diameter than the hole was placed into each hole, with its head secured under the hose clamp, to prevent the tag from sliding. The deployment process took on average 3 min. When the tag was attached, the drug reversal agent, naltrexone, was injected into the epidural vein. The animals were then intubated with a silicone endotracheal tube by manual palpation of the trachea. The cuff on the tube was inflated, and the animal was ventilated with 100% oxygen until it commenced breathing on its own. Once the animal started to lift its head, the tube was removed. For more details on immobilization, see Ølberg, et al.^26^. The whole process from the initial darting until the walruses were breathing on their own with a logger on their tusk took on average of 15 min 23 s. During this period std. body length, tusk length, and tusk circumference at the base was measured and samples of blood, blubber, skin, and whiskers were collected. No fatalities occurred. All procedures and animal handling were approved by the Norwegian Animal Care Authority (Ref. 2013/36153-2) and the Governor of Svalbard (Ref. 2014/00066-2 and 2015/00218) and were performed in accordance with all relevant guidelines and regulations. We have followed and reported according to ARRIVE guidelines that are relevant when working with animals in the wild.

### Data preparation

Out of the 40 tags deployed 33 reported data to logging stations. The main purpose of the present study was to explore the inter- and intra-individual variation in seasonal behaviours of walruses, and therefore only tracking records that lasted at least one year were included (including one with duration of 363.5 days). As a result, data from 12 individuals were analysed further (Table 1). All data processing was conducted using ‘R’ version 4.2.1^28^.

**Table 1:** Tagging information and GPS data specifications collected from male walruses in Svalbard, Norway.

ID	Location	Std. body length (cm)	Tusk length (cm)	Tusk girth (cm)	Tusk volume (cm^3^)	Tagging date	End of transmission	Transmission duration (days)	Total number of positions	Days with positions	Mean no. of positions per day	% of logs without position	Median/max length of gaps (hr)
41080	Sletteøya	335	55	18	472.7	19.07.2014	25.09.2017	1164	20,411	1087	11	42	3.2/1033
41033	Sletteøya	350	50	19	478.8	23.07.2014	26.11.2018	1587	31,983	1570	13	36	3.0/111
41052	Sletteøya	330	45	22	577.7	23.07.2014	05.11.2015	468	9049	449	10	50	3.3/144
41040	Sletteøya	304	43	17	329.6	25.07.2014	19.08.2015	389	20,411	371	9	50	3.3/184
65296	Sarstangen	356	47	23	659.5	04.08.2015	02.08.2016	363	8117	364	8	66	4.0/38
65306	Lågøya	340	47	18	403.9	08.08.2015	04.08.2017	726	16,378	726	16	30	2.2/25
65285	Lågøya	383	38	25	630.0	09.08.2015	23.12.2016	502	11,184	502	16	30	2.2/28
65294	Lågøya	315	39	16	264.8	11.08.2015	30.09.2017	780	16,205	777	10	52	3.2/40
65284	Lågøya	361	39	20	413.8	11.08.2015	30.10.2016	446	9554	443	10	55	3.1/98
65256	Lågøya	294	22	19	210.7	12.08.2015	10.02.2021	2008	43,589	2008	14	34	2.5/28
65301	Lågøya	340	45	20	477.5	12.08.2015	19.10.2021	2260	42,744	2235	9	54	3.7/343
65238	Lågøya	355	44	20	466.9	12.08.2015	25.12.2016	501	9554	501	17	25	2.2/15

The mean number of positions per day is based only on days with positions. Std. body length of the walruses was measured from the snout to tip of tail in a straight line. Tusk volume was calculated based on the formula *V* = *1/3 π r*^2^* h* where *r* is the radius and *h* is the tusk length^49^.

The raw positional data were initially screened for extreme outliers (assessed visually), which were removed, and for occurrences of duplicated positions (two positions with duplicated time stamps) where the second position was removed. The resulting number of positions ranged from 9554–31,983 per animal (Table 1).

A common issue with tracking data from marine mammals is the irregularity in the timed measurements as they surface to breathe at irregular times. Given that a GPS position could be logged every hour if the walrus is at the surface, and using the maximum recorded dive duration for walruses in Svalbard of 47.1 min^15^, a “gap” in the data stream was defined as more than 2 h passing between two GPS positions. To estimate locations at regular intervals, we used the “fit_smm” function from the “aniMotum” package in R^29^ using a state space model framework. We estimated a position every 3 h using a max speed of 2 m/s as in Freitas, et al.^19^. We did not interpolate across gaps of more than 96 h between two obtained positions (11 occurrences).

### Data analysis

To explore core habitats and migration paths we calculated Time Spent in Area (TSA) on 3 × 3 km grids using the R package ‘trip’^30^. Essentially, we summed the time spent in each grid cell based on the entire tracking record for each individual.

### Seasonal migration between core areas

Previous tagging studies from Svalbard^15,19^ have shown that male walruses migrate between areas occupied in the summer (usually on land) and areas occupied in the winter (usually on ice). Migrations to winter habitats had to occur sometime during the period between December to April. The start of the winter migration was defined as being when an individual spent at least 14 days at a distance greater than a pre-defined individual threshold from their summer area. This threshold was calculated as the mean value of the distance from the tagging site to all 3 h interpolated positions of each animal. Any trips above the distance threshold but of less than 14 days in duration were pooled into the summer habitat. For instance, a few of the animals made short trips to the area they would later spend the winter in and then returned to their summer habitat before migrating to the winter area to stay there for 2–5 months. These shorter trips are henceforth referred to as “scouting trips”.

One animal (ID 65256) travelled much longer distances than the rest, resulting in a distance threshold of 283 km (for the other walruses this threshold ranged between 90–163 km). This threshold resulted in positions obtained from this animal north of Kvitøya in Feb-May as being classified as summer habitat. This area overlapped the winter habitat for many other animals, (and shown as winter habitat in Freitas, et al.^19^), hence the maximum distance threshold obtained from the other animals (163 km) was applied to this walrus (ID 65256).

To estimate the timing of migration between the two seasonal habitats, and the time spent in them, we defined migration as starting when the animal left a 10 km buffer zone around a core area (described below). The animal then had to pass the distance threshold (see above) and then enter a 10 km buffer zone around a new core area. The summer migration of ID 65301 in 2020 was excluded as an outlier, due to this animal spending a long time outside its identified summer core area, resulting in a migration time of 77.6 days (the second highest was 35.2 days). Timing of migration and distance covered during migration were calculated from the interpolated data set to account for any gaps during this time. The yearly cumulative distance travelled was however based on the raw GPS data set.

### Core areas

We defined seasonal core areas as their 50% utilization distribution (UD) within each season (see above). To calculate these UDs we used a bivariate kernel method over a grid of 500 m x 500 m and the reference bandwidth (href) from the R package ‘adehabitatHR’^31^. Scouting trips to the winter areas occurring during the summer were excluded from these calculations. Incomplete seasons were included if they contained at least one month of tracking data. To assess the spatial consistency of each individual between years (site fidelity), we calculated the overlap of seasonal areas between consecutive years using the overlap index PHR_i,i+1_, which measures the probability of seasonal habitat of year i + 1 being located within seasonal habitat of year i^32^. As PHR is not a symmetrical index (PHR_i,i+1_ ≠ PHR_i+1,i_), we only calculated the one-way probability of overlap, namely whether year i + 1 is located within year i. PHRs were calculated for both 50% and 95% isopleths to quantify site fidelity at different scales. To compare overlap between 50 and 95% UDs, the PHR values were rescaled to the [0, 1] interval by dividing them by the highest possible value (0.5 and 0.95, respectively). We also extracted the area size of the seasonal 50% UD, both for complete and incomplete seasons, and calculated a mean centroid of winter and summer areas based on a 50% minimum convex polygon of all positions within each summer or winter period. Only complete seasons were used for this calculation, except in the few cases where only incomplete summer period existed (ID 65285, ID 65284), then the centroid was based on the mean of the incomplete seasons.

### Environmental drivers

To explore environmental drivers in relation to walrus behaviour and core areas, we extracted the number of daylight hours, the bathymetry and the sea ice concentration at each position. Daylight hours were extracted using the R package ‘suncalc’^33^. Bathymetry was extracted from IBCAO (International Bathymetric Chart of the Arctic Ocean, version 4.1, 2021). Daily operational sea ice charts were downloaded from the Norwegian Meteorological Institute^34^. In some cases, daily files were missing. In these cases the closest date was then used. Sea ice concentration is given as polygons corresponding to categories. To facilitate calculation of concentrations, we used each category’s middle value: Open water (0–1/10ths) was given an ice concentration of 5%, Very Open Ice Drift (1–4/10ths): 25%, Open Ice Drift (4–7/10ths): 55%, Close Ice Drift (7–9/10ths): 80%, Very Close Ice Drift (9–10/10ths): 95% and Fast Ice (10/10ths) was given 100%. To calculate the average sea ice concentration in core area for each animal, ice layers were rasterised using the 'fasterize' package in R^35^ using a 0.05 × 0.05 decimal degree resolution. Daily ice values were extracted using the ‘terra’ package^36^.

To examine the effect of sea ice as a possible driver for migration, we calculated the daily mean ice concentration based on the animal’s positions in the 14 days before the defined time of migration. We tested whether the daily sea ice concentration differed between two weeks (14 to 8 days) prior to migration and the week (7 to 1 days) prior to migration using a mixed effect model (R package ‘nlme’,^37^). ID was included as a random effect to account for multiple measurements from the same animal. We also included an autocorrelation term and a weighted structure (constant variance function), allowing the variance to differ between years. Initial data exploration revealed large differences in ice concentration in the summer habitats between animals tagged in the north and in the south, hence, these two groups were tested separately. For the test on animals tagged in the north (Lågøya), ice concentration was arcsine square root transformed for a better model fit.

The effect of environmental drivers (depth and ice concentration) on walrus behaviour was examined by means of the time varying move persistence parameter γ_t_, in a mixed effect model using the ‘mpmm’ package in R^38^. This index ranges continuously from 0 to 1 and is based on the correlation in step lengths and turning angles between regularized locations of a track. This index represents changes in movement patterns with low move-persistent values corresponding to low correlation between successive locations reflecting area restricted behaviour often associated with foraging (or breeding). Conversely, high move persistent values correspond to high correlation between successive locations indicating directed movement associated with transiting^29,38^. Values of γ_t_ were modelled in a mixed effect linear framework as a function of ice and depth, where depth was log-transformed. Due to possible differences in seasonal habitat selection and the lack of framework for including factors or interactions in the model, we used separate models for winter and summer. We specified each year as separate tracks (“tid” identifier) for each individual. Ice and depth were allowed to vary per individual to assess individual variability in habitat use. Nine candidate models were evaluated as combinations of the full model:

$$logit({\gamma_t}) = {\text{ice}} + {\text{depth }}\left( {{\text{ice}} + {\text{depth}}\left| {{\text{id}}} \right.} \right)$$

Model selection was based on differences in AIC and likelihood ratio (LR).

## Results

### Tag performance

**Figure 3:**
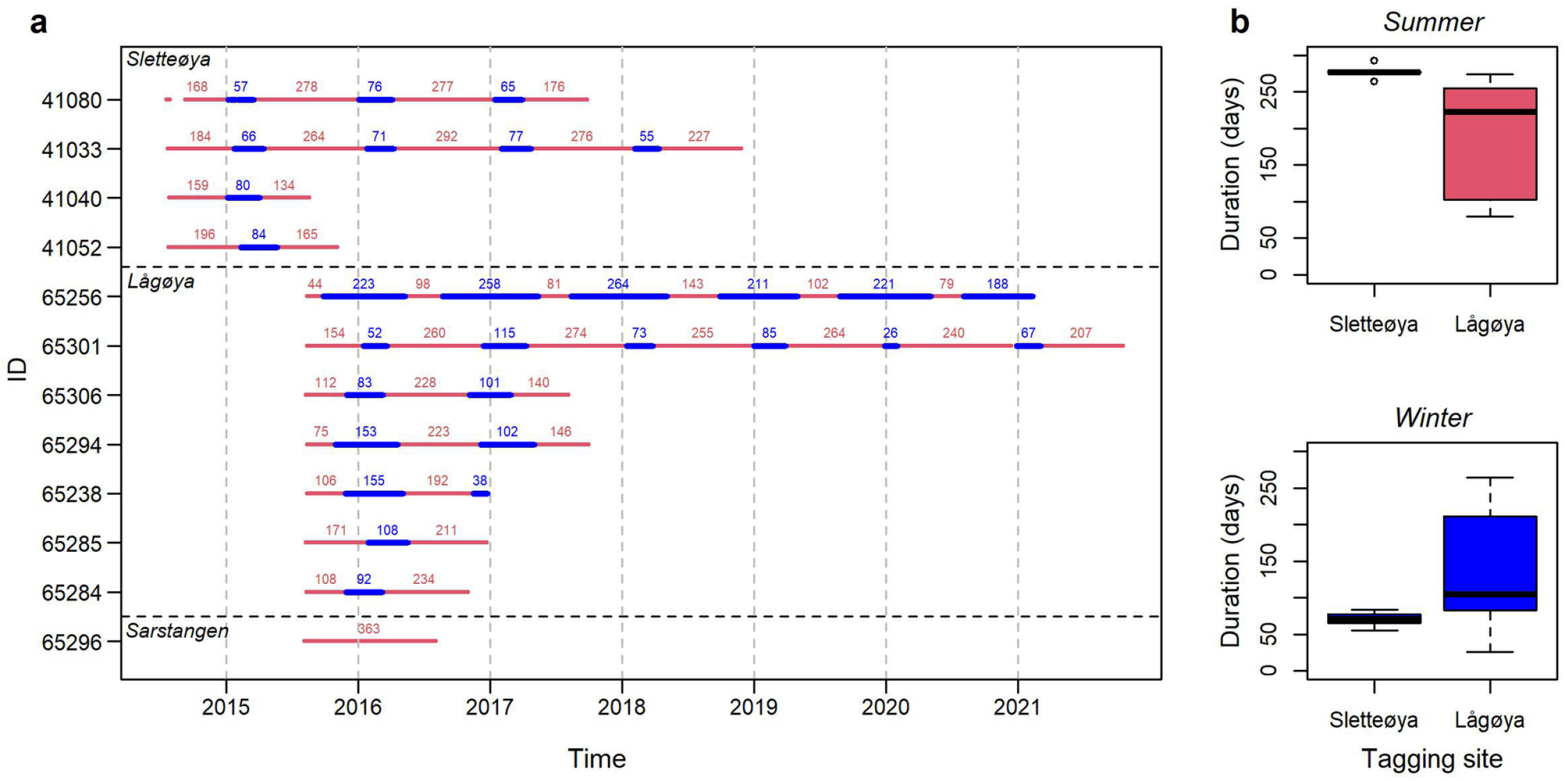
Tracking durations for 12 male walruses instrumented on Svalbard in 2015 and 2016. (**a**) time spent in the wintering areas (thick blue lines) and in summering areas (red lines). Numbers above the line indicate the number of days spent in each seasonal areas. (**b**) comparison of duration of stay in the summer and winter areas based on tagging location.

Track duration of the twelve individuals included in the study ranged from 363 to 2260 days (1–6.2 years, Table 1, Fig. 3a) with an average of 933 days (SD = 668). Not all days in the tracking period contained positions, but at least one position was obtained in 93–100% of all days and on average 12 positions (8–17, Table 1) were obtained per day with at least one GPS position. The tags did not manage to fix a GPS position at every attempt, 25–66% of all attempts resulted on only time being logged (Table 1). Hence, gaps between consecutive GPS positions occurred regularly throughout the datasets. The longest gap between two positions was 1033 h (43 days) (Table 1, Fig. 3), but most gaps were of short duration (median gap length 2.2–4 h, Table 1).

### Migration characteristics

**Figure 4:**
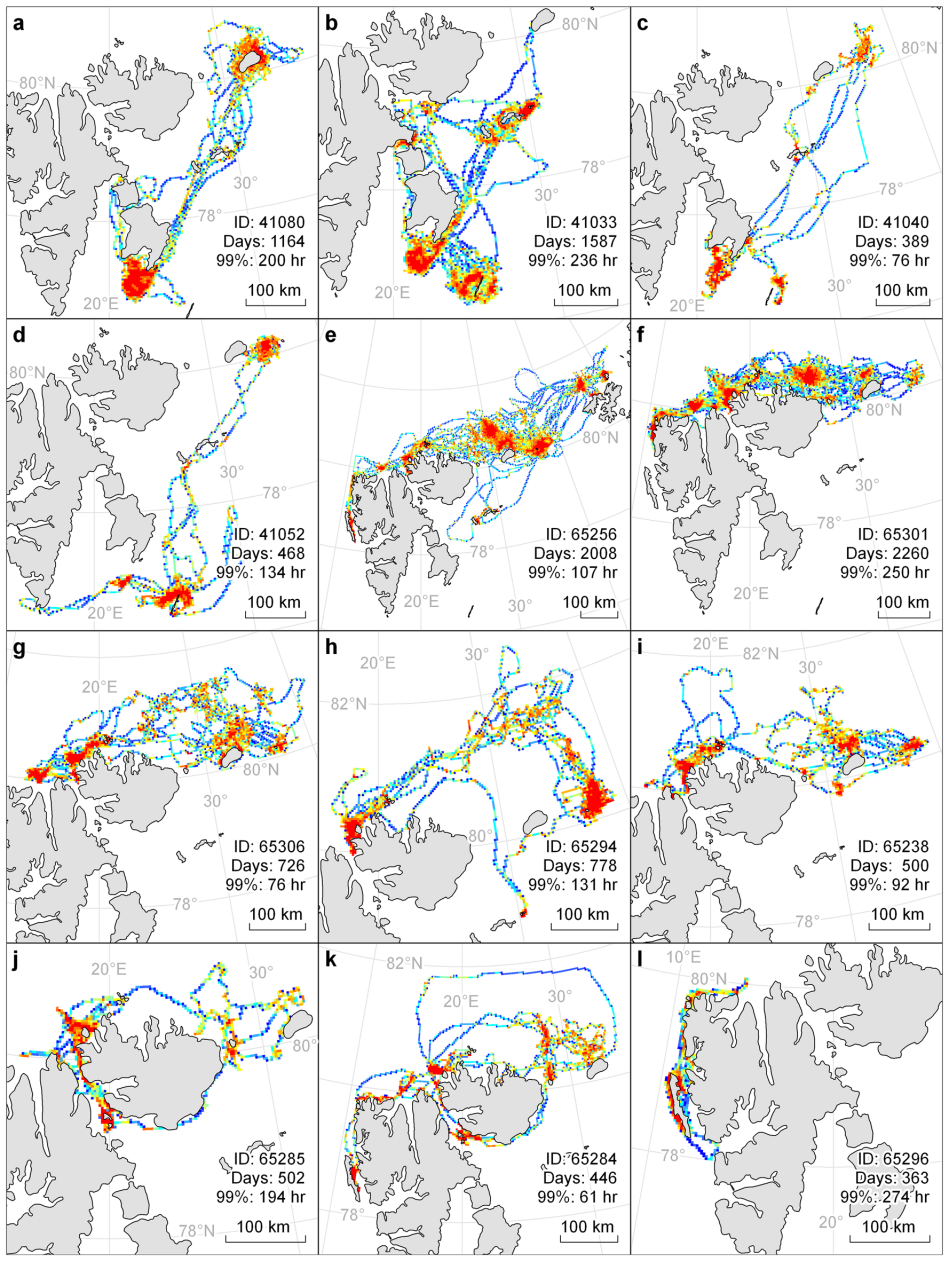
Time spent in area (TSA) by male walruses in Svalbard, Norway. TSA values are displayed as percentiles of the distribution of the values for each individual. Warmer colours reflect the highest percentile of the distribution. For each plot the total number of tracking days is noted as well as the 99th percentile value of TSA (in hours). Tagging location: (**a**–**d**) Sletteøya, (**e**–**k**): Lågøya, (**l**): Sarstangen. The maps were generated in Esri ArcMap v10.8.1, https://desktop.arcgis.com/en/arcmap/index.html.

**Figure 5:**
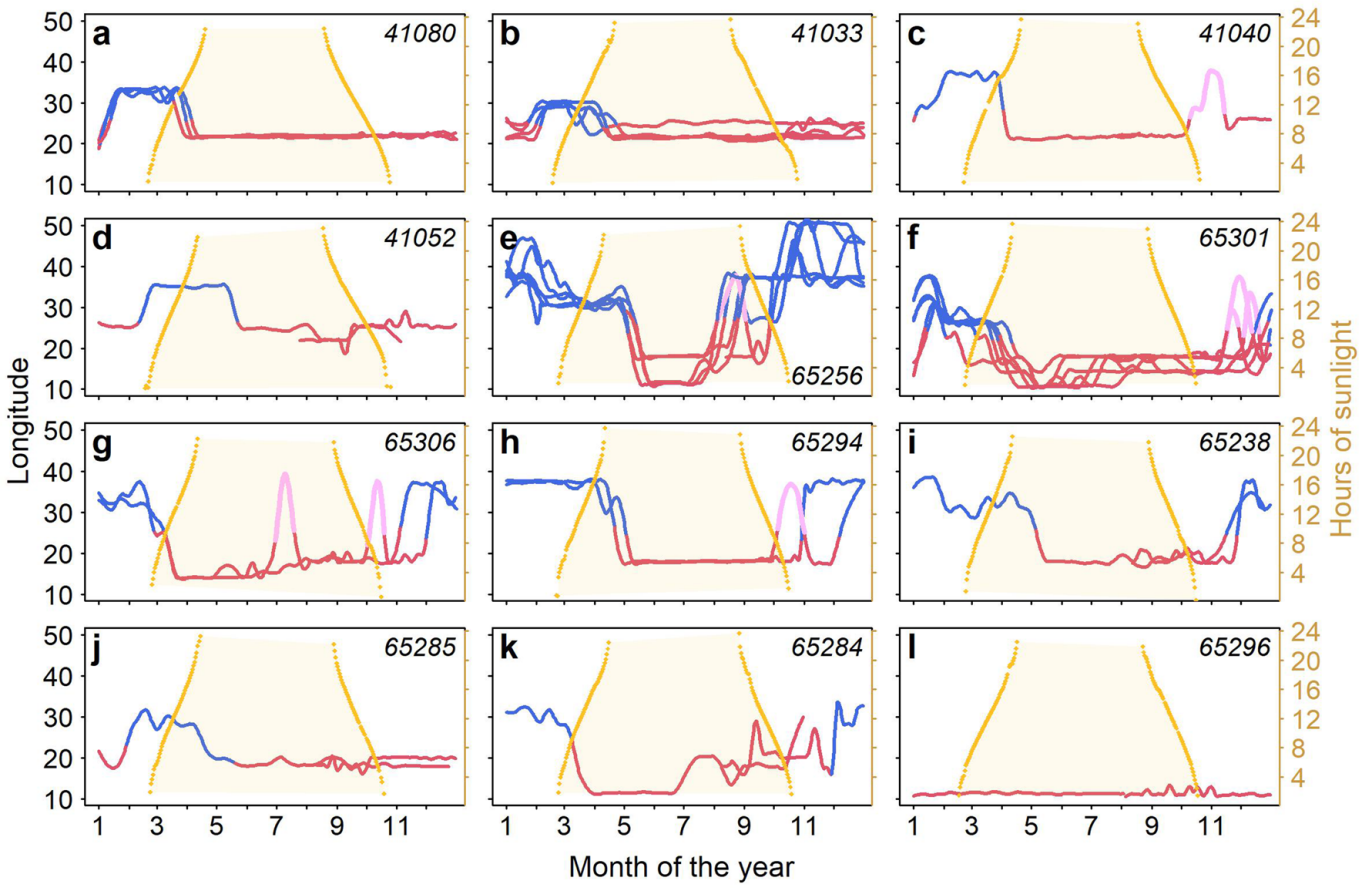
Longitudinal positions for 12 male walruses tagged in Svalbard, Norway vs daylength. Colours indicates the season, red for summer and blue for the winter. Scouting trips are marked in light purple. The number of daylight hours are plotted in yellow on the right axis, based on the individual animal’s locations in the first year of tagging.

Eleven of the 12 tagged individuals exhibited seasonal migratory behaviour, moving significant distances away from the summer area where they were instrumented, during the winter period (Fig. 4, 5). The non-migratory individual had the shortest tracking duration and was the only animal instrumented at Sarstangen on the west side of Svalbard (Fig. 4l). Regardless of their tagging locations, all migratory individuals moved in a northeast (Fig. 4a–d), or easterly direction (Fig. 4f–k) and 10 individuals spent the winter between Nordaustlandet and Franz Joseph Land above 80° North. The last individual migrated in the same general direction as the others but spent four consecutive winters further south, in the vicinity of Kong Karls Land (Fig. 4b).

**Figure 6:**
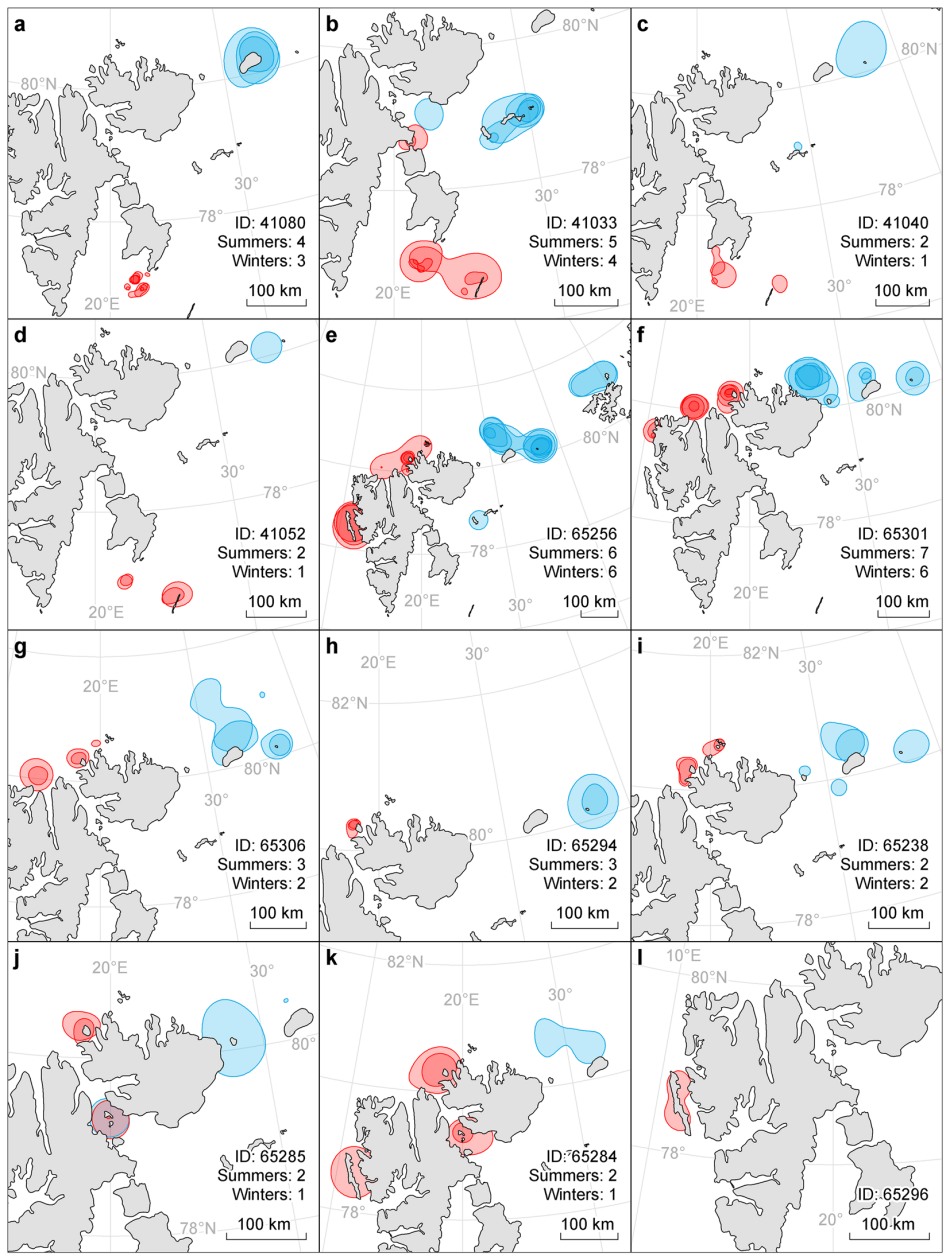
Core areas occupied by 12 male walruses in Svalbard, Norway. Core areas are based on 50% utilisation distribution. Red indicates summering areas and blue indicates wintering areas. The total number of seasons included for the specific animal is given in the text. Tagging location: (**a**–**d**) Sletteøya, (**e**–**k**) Lågøya, (**l**) Sarstangen. Notice that the distance indicator varies between plots. The maps were generated in Esri ArcMap v10.8.1, https://desktop.arcgis.com/en/arcmap/index.html.

The summering areas were more widely spread out than the wintering areas. The four individuals tagged at Sletteøya, in the south of Svalbard (Fig. 1), all returned to the south each summer, to areas around Tusenøyane and Hopen (Figs. 4a–d, 6a–d). Their migration paths followed direct routes from Kvitøya/Victoria Island to Kong Karls Land and to the eastern coastline of Edgeøya or towards Hopen in more open waters. Migration duration varied between 6 and 17 days (mean = 10, SD = 4) and the migration distances ranged between 226–665 km (mean = 416, SD = 108). The distance between individuals’ summer and winter core areas, ranged between 256 and 425 km (mean = 366, SD = 75).

The seven walruses tagged at Lågøya, in the north of Svalbard (Fig. 1), all returned west in the summer, mainly staying in the northern parts of Svalbard (Figs. 4e–k, 6e-k). Migration paths between winter and summer areas varied between being relatively coastal, with animals travelling along the north coast of Nordaustland (Fig. 4f), to more offshore routes (Fig. 4h,k). Migration duration varied between 1 and 35 days (mean = 10, SD = 9) and migration distances ranged from 93–1066 km (mean = 370, SD = 264). Distance between seasonal core areas ranged between 171 and 401 km (mean = 274, SD = 73).

The yearly cumulative distances travelled for all animals ranged between 5225–10,406 km, with a mean of 7893 km (SD = 1308) in the first year of tracking (Table 2). However, at the intra-individual level, the cumulative distance travelled varied relatively little between consecutive years, with a mean difference of 427 km (27–1196 km, SD = 390, Table 2).

**Table 2:** Yearly cumulative distances travelled by walruses equipped with GPS loggers in Svalbard, Norway, in 2014–2015.

ID	Year 1	Year 2	Year 3	Year 4	Year 5	Year 6
41080	7209	7955	8357			
41033	8196	7154	7127	6263		
41040	7540					
41052	10,137					
65256	9284	9484	9540	8822	8541	
65301	6465	6802	6494	6569	6475	6314
65306	10,406	9210*				
65294	7661	8283				
65238	8801					
65285	5225					
65284	8350					
65296	6731**					

A whole year is defined from the time of instrumentation (except for ID 41080, where the start time was set to 9 Aug, due to a 43-day gap in the beginning of the data set).

*Cumulative distance based on 360 days. **Cumulative distance based on 363 days.

The timing of migration to the winter areas varied considerably between individuals (Fig. 5). Animals tagged at Sletteøya in the south of Svalbard (Fig. 5a–d) left their summer core areas between 29 Dec–6 Feb, which was well into the Polar Night and even close to the return of daylight in one case (ID 41052, Fig. 5d). These walruses stayed between 55–84 days (median 71) in their winter core habitat (Fig. 3b). For most animals tagged at Lågøya in the north (Fig. 5f–k), migration to winter areas occurred between 27 Oct–13 Jan, 1–2 months earlier than for animal tagged in the south. They stayed 26–264 days (median 105) in their core winter habitat (Fig. 3b).

The intra-individual timing of winter migration was consistent between years. For walruses tagged at Sletteøya in the south, the annual intra-individual variation in migration timing was on average 10 days (5–17 days, SD = 5, Fig. 5a–b). For walruses tagged at Lågøya in the north, the annual intra-individual variation was on average 30 days (9–48 days, SD = 11, Fig. 5e–i).

Five animals made early, short trips to the winter areas (scouting trips), returning to their summer area for a period before again migrating to their winter area for an extended period of time (light purple, Fig. 5c,e–h). These individuals were among the youngest animals based on tusk volume at the time of tagging (210.7–477.5 cm^3^, Table 2).

There was a lot of variation in the timing of individual returns to the summering grounds but none of the animal left the winter area before the return of daylight. The migration to summering grounds happened when daylength reached approximately 12 h, in March–April, for approx. half of the animals (Fig. 5a–c,f,g,k), whereas the other half returned during the midnight sun period in May (Fig. 5d,e,h–j). Individuals returned to their summer ranges at relatively consistent times over multiple tracking seasons (annual intra-individual variation being 1–60 days, mean = 15, SD = 15, Fig. 5a–b,e–h).

### Site fidelity

**Figure 7:**
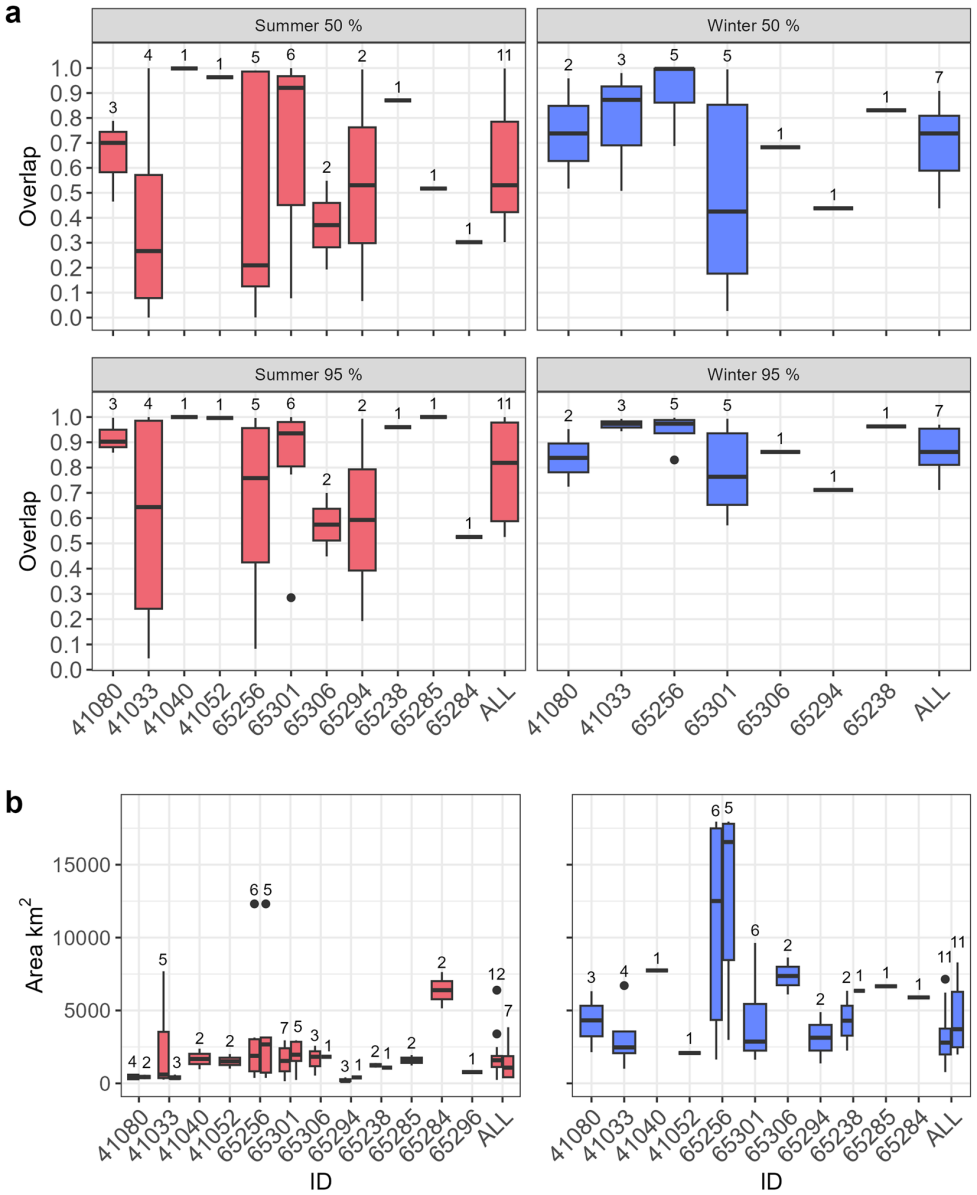
Site fidelity and area size of seasonal home ranges of male walruses in Svalbard, Norway. (**a**) Spatial consistency measured as the year-to-year overlap of 50% and 95% utilization distributions using the PRH index. The number of overlap values included in each boxplot is indicated above each box. The ALL value is based on the mean of individuals (with the number of individuals included on top). (**b**) Area of 50% utilization distributions for summer (red) and winter (blue). Two boxplots are given per ID if incomplete seasons could be excluded. Hence, in the first boxplot all seasons are included and in the second only complete seasons if min. one full season remained. Numbers of seasons in each animal’s data set are given above each box.

All individuals exhibited clear site fidelity to previously used areas (Fig. 6). The overlap in interannual 95% utilisation distributions for animals with multiple seasons suggests very strong seasonal site fidelity both in summer and winter (80–85%, Fig. 7a). Overlap of core areas, defined as the 50% utilization distribution areas, was on average 55% between years in summer and 75% overlap on average in winter (Fig. 7a), though it must be noted that there were large variances because of the strong individual patterns displayed by the animals.

The size of core areas (50% UD) was in general larger in the winter (~ 3700 km^2^, only complete seasons, Fig. 7b) than in summer (~ 1000 km^2^, only complete seasons, Fig. 7b). This was especially evident for ID 65256 who had the largest wintering area. This animal was the only individual that travelled westward all the way to Franz Josef Land (Fig. 4e); it was also the youngest individual based on tusk volume (Table 1).

### Environmental characteristics of seasonal habitat

All core areas were located on the continental shelf. However, summer core habitats were characterised by shallower water depths (median 18–89 m, Supplementary Fig. S1) compared to winter areas (median 91–144 m).

**Figure 8:**
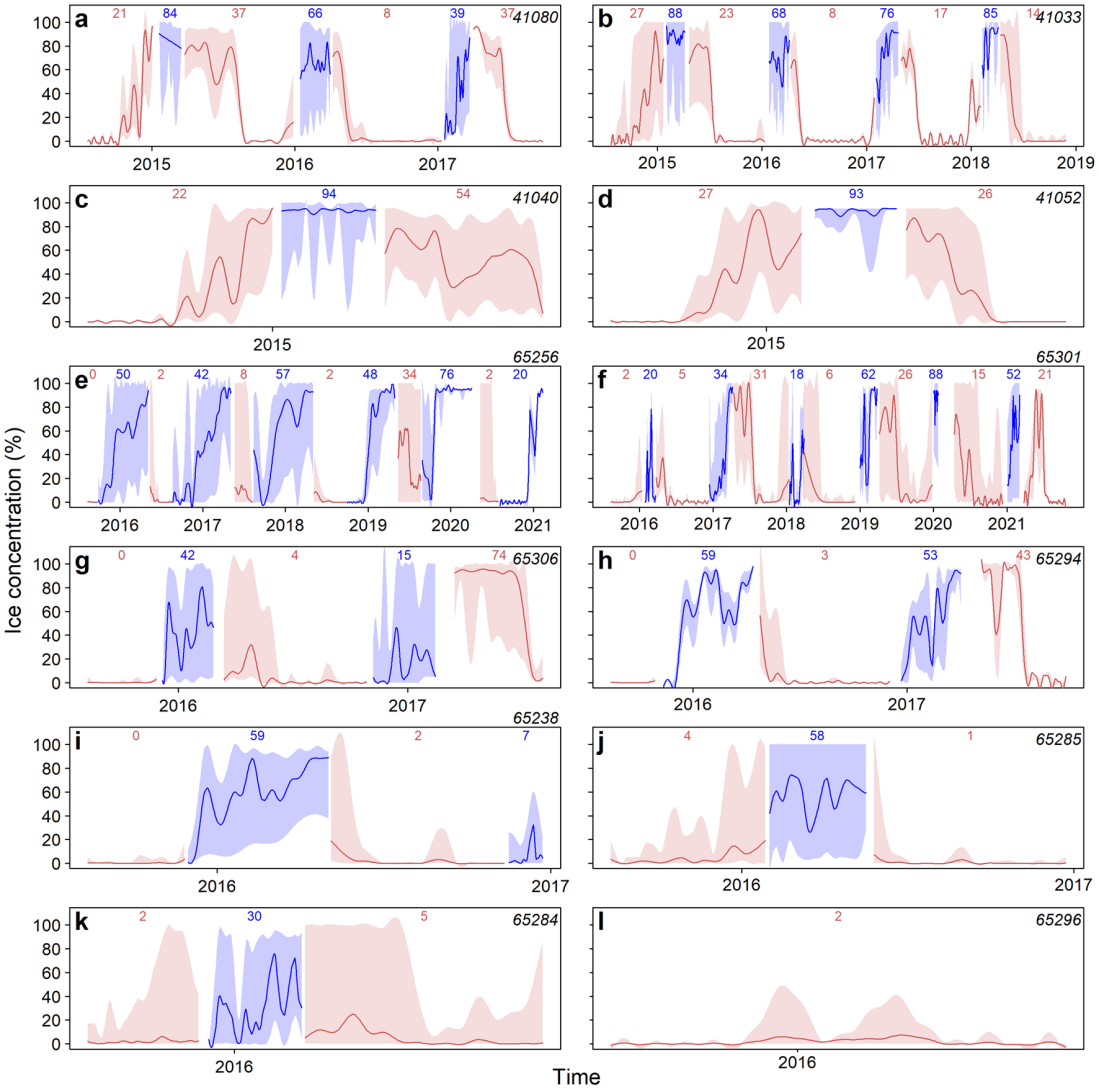
Average daily sea ice concentration in core areas of male walruses in Svalbard, Norway. Daily ice concentrations in kernel areas were extracted from a 0.05 × 0.05 decimal degree analysis grid of operational sea ice charts. The colours indicate the season, red for summer and blue for winter. The shaded area behind the lines illustrates the range of ice concentrations encountered each day within core areas (50% UD). Above each season the mean sea ice concentration is given. Animal ID is inserted in the top right corner of each panel.

Sea ice concentration varied considerably between years and between core areas, but the winter areas generally had higher sea ice concentrations compared to summer areas (Fig. 8). Summer core areas were ice-covered at the start of the summer regardless of their location but were largely ice-free by the end of the summer season.

Sea ice concentrations in winter core areas of animals tagged in the south ranged from 39–94% coverage (Fig. 8a–d). Animals tagged in the north (Fig. 8e–k) experienced highly variable sea ice concentrations during their winter periods, ranging from 16 to 88%.

### Drivers of migration

Changes in sea ice concentration were not linked to the departure date of migrations to the winter grounds. There were no significant differences in sea ice concentration along the tracks of the walruses between two weeks prior vs one week prior to winter migration (Table 3). Sea ice concentration was however considerably lower along the tracks of animals migrating from Lågøya, in the north of Svalbard, compared to sea ice concentration along the tracks of animals migrating from Sletteøya in the south (Supplementary Fig. S2).

**Table 3:** Model coefficients for linear mixed effect model of ice concentrations before winter migration.

	Predictor	Value	SE	df	t-value	*p*-value
Tagged at Lågøya	Intercept	0.114	0.040	236	2.855	0.005
	Year 2017	− 0.095	0.037	236	− 2.551	0.011
	Year 2018	0.235	0.108	236	2.187	0.030
	Year 2019	− 0.067	0.073	236	− 0.924	0.356
	Year 2020	0.286	0.162	236	1.772	0.078
	Year 2021	0.060	0.067	236	0.892	0.373
	Period	− 0.006	0.015	236	− 0.371	0.711
Tagged at Sletteøya	Intercept	57.873	11.028	118	5.248	0.000
	Year 2016	− 59.669	8.001	118	− 7.458	0.000
	Year 2017	− 43.697	11.193	118	− 3.904	0.000
	Year 2018	− 25.147	13.297	118	− 1.891	0.061
	Period	8.627	5.036	118	1.713	0.089

The model was tested on two periods 14–8 vs 7–1 days before migrating to the wintering areas and was applied separately for the walruses tagged at Lågøya (northern Svalbard) and at Sletteøya (southern Svalbard).

For all the animals, the sea ice concentration was generally high in the weeks prior to the onset of migration towards the summering areas (Fig. 8, Supplementary Fig. S2). Differences in ice concentration were not tested among the two weeks prior to the onset of summer migration due to poor model fit.

### Habitat use

**Figure 9:**
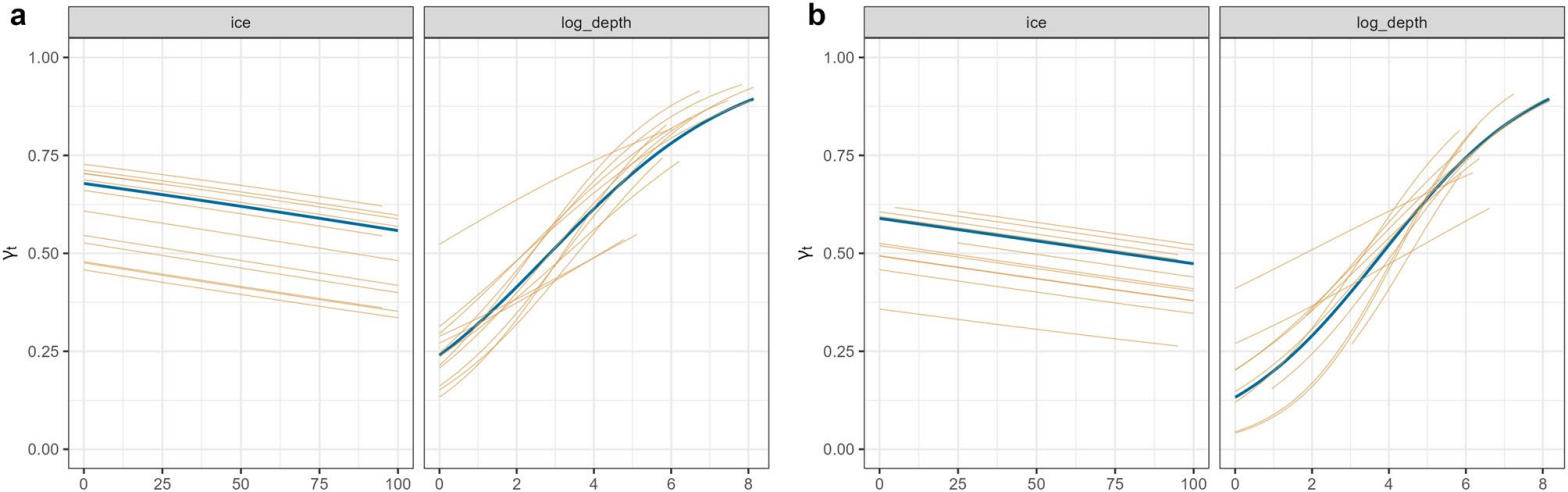
Environmental drivers for male walruses’ behaviour in Svalbard, Norway. Graphs are based on results of the most parsimonious move persistence mixed effect model for the summer (**a**) and winter (**b**) seasons. The panels illustrate individual random effects relationships (brown lines) and fixed effects (blue line) responses to ice concentration (0–100%) and depth (notice the logarithmic scale, larger values indicate deeper depths). For both seasons the most parsimonious model is represented by *logit*(γ_t_) = ice + depth + (depth | id).

Movements of walruses represented by the move persistence index γ_t_ were analysed for the summer and winter seasons separately. For both seasons the most parsimonious model included both ice and depth as fixed terms and allowed for random slopes for depth (Table 4). Model results showed a clear tendency for more tortuous movements (low move persistence) in shallow areas and more directed movement (higher move persistence) in deeper areas in both seasons (Fig. 9). There was also a tendency for more tortuous movements when ice concentrations were high, in both summer and winter.

**Table 4:** Model ranking of the move persistence mixed effect models based on tracking data for male walruses tagged in Svalbard, Norway.

Season	Model formula	df	ΔAIC	ΔLR
Summer	~ ice * depth + (depth | id)	**7**	− **423,976.4**	**211,995.2**
	~ ice + depth + (1 | id)	5	74.49	39.25
	~ depth + (depth | id)	7	184.52	92.26
	~ depth + (1 | id)	5	253.93	126.96
	~ ice + (ice | id)	7	1062.31	573.99
	~ ice + (1 | id)	5	1143.98	531.15
	~ 1 + (1 | id)	5	1164.06	585.03
Winter	~ ice * depth + (depth | id)	**7**	− **163,578.2**	**81,794.83**
	~ ice + depth + (ice + depth | id)	10	8.50	1.25
	~ ice * depth + (1 | id)	5	76.47	40.23
	~ ice + depth + (ice | id)	7	77.56	38.78
	~ depth + (depth | id)	7	123.15	61.57
	~ depth + (1 | id)	5	199.08	101.54
	~ ice + (1 | id)	5	874.96	439.48
	~ ice + (ice | id)	7	875.14	437.57
	~ 1 + (1 | id)	5	950.24	477.12

Separate models were applied to winter and summer. Model ranking is based on changes in the Akaike information criteria (ΔAIC) and likelihood ratios (ΔLR). The most parsimonious models, indicated in bold, show the AIC (not ΔAIC) and LR (not ΔLR) values. Models that did not converge are now shown.

## Discussion

Successful acquisition of long-term positional data sets for Svalbard walruses was accomplished with tusk-mounted, custom-designed GPS tracking devices. Out of 40 tag deployments, 12 tags transmitted data for more than one year and in six cases the transmission records lasted between two and six years. This is, to our knowledge, the first time that positional data have been obtained for any marine mammal for six consecutive years based on a single deployment event.

Obtaining long term tracking data is a compromise between acquiring detailed data on dives and haul out behaviour and longevity of the tag containing a single D-cell battery. The tags developed for use in this study transmitted only position data to maximize the lifetime of the tag. The two longest data sets—2008 days (5.5 years) and 2260 days (6.2 years)—exceeded the expectation of the battery-life (approx. 5 years).

In seven of the 40 deployed tags, no data were received at all and for 21 tags the data collection took place for 1–162 days. Whether these short, or null, data sets were due to tag failure, tag loss, or these animals not returning to an area with a logging station is not known. The tags must come within approx. 500 m of a receiver station for the data to be transmitted/downloaded. However, walruses are known to be quite rough on deployed instruments, and hence, early transmission stops could be due to instruments breaking or falling off. One of the tagged walruses (ID 65296) was observed with the tag missing the lower clamp and one animal was observed with just a hose clamp hanging loose on the tusk (C. Lydersen pers. obs. a few summers after the deployments). Still, as demonstrated here and in earlier studies^19,21^, tusk deployment is the most robust deployment method to collect long-term tracking data from walruses.

The tags were programmed to acquire a GPS fix every hour when the animal was at the surface. Yet, across the 12 data sets analysed in detail herein, only the time of a GPS attempt was logged and not a position in 25–66% of all sampling hours. The reason for unsuccessful position acquisition could be low satellite coverage (a constellation of minimum four is needed to acquire a meaningful position) or the sky-view being blocked in some manner (the animal lying on top of the tag or other animals being in the way). Some long periods without a GPS fix did occur while the walruses were migrating over long distances. Hence, the position of the tag on the tusk likely also affects successful position fixing while animals are swimming. Walruses often swim at the surface without exposing their tusks when travelling, whereas when they are foraging, they tend to come up more vertically to breathe at the surface before diving again (C. Lydersen pers. obs.). Hence, both behavioural aspects and varying satellite coverage likely influence the rate at which successful GPS positions are obtained by tusk-mounted tags.

All walruses in this study showed clear site fidelity to previously used summer and winter habitats in consecutive years. It was assumed that the tagging sites were within summer feeding grounds and that migrations were undertaken to the winter/breeding grounds every year. Such directed movements were performed seasonally by all but one animal tagged on the west coast of Spitsbergen. After the winter, all migrating animals returned to the same general area in which they had been tagged in the summer and the multiyear data sets demonstrated a return to same wintering grounds for several consecutive years. While general utilisation distributions (95% UD) were largely overlapping, the core areas (50% UD) shifted for some animals between two or three areas. Such shifts may reflect a response to shifting prey abundance or a response to interannual sea ice variability. The interannual overlap in area usage was in general larger in winter than in summer, which in turn may indicate that male walruses target specific areas where females are known to occur.

The distinct fidelity to previously occupied areas by male walruses in Svalbard was documented by Freitas, et al.^19^, where the data allowed for analysis of overlap in the month of August between two consecutive years for five individuals. In four out of five animals, there was considerable spatial overlap in area usage after returning from their winter grounds. In NE Greenland, a single walrus was caught several times and tracked during four seasons in 1989, 1990, 2000 and 2001^17^. This individual showed clear homing behaviour, returning to the same haul-out site and using the same foraging grounds each summer. This animal followed an almost identical migration path each year and it appeared to use the same polynya each winter. It also arrived and departed at consistent times every season and its movements were relatively independent of annual variations in ice and temperature regimes. Thus, strong fidelity to previously occupied areas seems to be a general trait for walruses and they appear to be robust to dynamic environmental variables such as sea ice.

There was consistency among animals in their departure times from the winter areas in spring, while the timing of migration from the summering areas to wintering areas was more variable. This was especially the case for animals conducting scouting trips to the winter areas. Wiig et al.^20^ suggested that some long-range, round-trip movements by male walruses from southern areas of Svalbard, observed during autumn, were not directly connected with breeding activity, but rather a sort of reconnaissance in search of females. Jay and Hills^21^ suggested that similar behaviour existed in walruses from the Bristol Bay, Alaska, where some individuals travelled to their breeding area as early as November, although breeding does not occur until January. Only one individual in Freitas, et al.^19^ conducted such an early season trip. In the present study, five out the twelve animals undertook such roundtrips to their winter grounds before spending significant periods in them.

Lowther, et al.^15^ inferred that male walruses in Svalbard initiate breeding migrations close to the onset of the Polar Night and return to foraging grounds towards the peak of the midnight sun period. This pattern was not consistently the case for all animals in the present study, where individuals tagged in the south of Svalbard initiated migration to winter (breeding) habitats one-two months into the Polar Night and in one case even close to the return of the sun (ID 41052). In general, animals tagged at Sletteøya in the south migrated one-two months later than animals tagged at Lågøya in the north, and although daylight disappears approximately 10 days later in the south of Svalbard, the difference in day light is not sufficient to explain the difference in the timing of migration. Other factors such as the advance of sea ice formation may explain such differences. Both in Greenland and Alaska, abandonment of foraging grounds and coastal haul-out sites is prompted by the development of sea ice formation^16,17,21^. Sea ice occurs earlier in the north of Svalbard, compared to the south. However, for animals tagged at Lågøya (north), there was little or no sea ice present in their summer habitats preceding the winter migration, likely because of the earlier departure undertaken by the animals in the north. Some sea ice was present at the time of migration for animals tagged at Sletteøya (south), although we did not find a significant increasing trend in sea ice immediately prior to migration.

In general, it seems that individual animals have different space use strategies from one another. Differences may be linked to age and experience. Indeed, we found that the youngest animals performed scouting trips, suggesting that they lack experience in locating females at the right time. We also found that the youngest animal (ID 65265) migrated much earlier in the year and was the only one that that travelled all the way to Franz Josef Land (mainly in the first years of his data record). In Freitas, et al.^19^, a few of the animals tagged in the south of Svalbard did not migrate in winter, and one individual went to the southwest coast of Spitsbergen in winter. Freitas, et al.^19^ speculated that these animals were relatively young (based on tusk volume). Yet, in the current study, animals that were of similar size to those studies by Freitas, et al.^19^ showed clear migration patterns. Which parameters drive the different migration strategies is not entirely clear.

The oldest animal based on tusk volume (ID 65296) was the only animal that did not migrate during the winter. This was also the only animal tagged at Sarstangen, on the west side of Svalbard. This area contains far fewer animals in the winter compared to the east side of the archipelago. However, with the increasing number of walruses in Svalbard, more females are now also being observed here (C. Lydersen pers. obs.), potentially making it an attractive site to stay in winter for an old male, where competition from other males might be low.

Identified winter habitats were somewhat overlapping with the winter areas identified in Freitas, et al.^19^, with a clear hot spot north of Kvitøya and Victoria Island. It is presumed that the walruses migrate to these areas to find females as Atlantic walruses mate between January and April^12,13^. Calves have been spotted in these areas in summer in recent years^11^. Lowther, et al.^15^, showed that male walruses that migrated to these offshore sites in winter did more shallower dives in this period, with almost no benthic diving, suggesting that little foraging took place and that the dive behaviour was likely associated with mating activity, as described by Sjare and Stirling^13^.

Ice concentrations in the winter areas varied significantly between years, ranging from 16 to 94% across years and areas. The walruses associated with a specific area, as opposed to a specific ice concentration. The move persistence mixed model showed a tendency for more tortuous movements at higher ice concentrations, which might suggest that heavy ice is important for breeding and as resting platform, and that this is where females are found in winter. Alternatively, the walruses might have to navigate a more complex path to find open areas for breathing in heavy ice. Atlantic walrus females have been shown to be closely associated with ice^13^, but they must also select wintering areas that provide sufficient access to open water and predictable food resources. As discussed in Lowther, et al.^15^, the wintering areas found around Kvitøya and Victoria Island are likely associated with persistent polynyas^39,40^ that would allow female walruses to find enough food resources and yet have reliable ice in winter for hauling out. However, the winter core areas found here were often more than 100 m deep. If female walruses are indeed foraging in this area, it is not known what type of prey they might be targeting at these depths.

Summer habitats were very coastal and occurred in shallow areas. Movements were clearly more restricted in shallower waters, indicative of foraging and resting. The strong fidelity to previously occupied summer habitats suggests that food resources in these areas are predictable and likely abundant.

The Svalbard walrus population has recovered markedly since their protection in 1952, when only a few hundred individuals remained at a handful of sites^9^. Surveys indicate an increasing presence of female walruses with calves and previously utilized sites are being reclaimed as the walrus population expands across the archipelago^11^. These positive developments are occurring despite a likely overall decrease in the region's capacity to support walruses, owing to declining sea ice and concomitant impacts on the benthic community^41^. Historical records indicate that the animals in Svalbard were 33% females (at Tusenøyane) 100 years ago^42^. If females are increasingly returning to the Svalbard area, future migration patterns of males will likely change, although both sexes might migrate between summer and winter areas.

Although the population show signs of recovery, climate change is occurring in the Arctic at an unprecedented rate, with inevitable effects on walruses in Svalbard, given their dependence on sea ice as a winter breeding and resting platform^3^. Breeding females are likely also dependent on sea ice for protection of their calves during winter storms and for protection from open water predators such as killer whales (*Orcinus orca*). Hence, if sea ice recedes further and further north during winter (breeding time), the females are likely to follow it until the shelf edge is reached. At some point when sea ice only extends over the deep Arctic Ocean, they will no longer be able to forage in the vicinity of the winter ice. Svalbard walruses might be forced to rest and breed on land, which will limit their foraging range and force females to increasingly use terrestrial haul out sites, similar to what has already been observed in the Pacific Arctic^43,44^. Massive onshore, mixed sex haulouts in the Pacific population have recently experienced increased mortality of young walruses due to crushing during rushes to the sea^43,44^.

Loss of sea ice will also have indirect effects on walrus ecology, via effects on pelagic-benthic coupling whereby the vertical flux of sympagic fauna supports benthic production^45,46^, thus potentially reducing the prey-base for walruses. The increasing warming and increased inflow of Atlantic water is additionally altering the distribution and abundance of prey species in the Arctic ecosystem, Atlantifying the northern Barents Sea region^47^. All of the concomitant changes may force walruses to adjust their foraging patterns and might result in reduced prey abundance.

While strong site fidelity may be linked to reproductive successes and survival^48^, disruption of traditional breeding sites and foraging habitats may impact the viability of the population if it is unable to adapt fast enough. Hence, strong site fidelity can make the walruses vulnerable to changes in their ecosystem. Continuous climate warming and the impact on the Arctic ecosystem may affect the predictability of the resources that are vital to walruses. Therefore, continuous monitoring of walruses in Svalbard will be important in coming years to assess their adaptability to environmental changes. Long-term tracking offers a valuable opportunity to study their behavioural flexibility in response to shifting ecosystems.

## Supplementary Information

Supplementary Figures.

Supplementary Information 2.

## Data Availability

All data generated or analysed during this study are included in this published article [and its supplementary information files].

## References

[1] Wassmann, P., Duarte, C. M., AgustÍ, S. & Sejr, M. K. Footprints of climate change in the Arctic marine ecosystem. *Glob. Change Biol.* **17**, 1235–1249 (2011).

[2] Born, E. W. *et al.* Underwater observations of foraging free-living Atlantic walruses (*Odobenus rosmarus rosmarus*) and estimates of their food consumption. *Polar Biol.* **26**, 348–357 (2003).

[3] Fay, F. H. Ecology and Biology of the Pacific Walrus, *Odobenus rosmarus divergens Illiger*. *N. Am. Fauna* **74**, 1–279 (1982).

[4] Lindqvist, C. *et al.* The Laptev Sea walrus *Odobenus rosmarus laptevi*: an enigma revisited. *Zool. Scr.* **38**, 113–127 (2009).

[5] Lydersen, C. Walrus: *Odobenus rosmarus* in *Encyclopedia of Marine Mammals (Third Edition)* (ed. Würsig, B., Thewissen, J. G. M. & Kovacs, K. M.) 1045–1048 (Academic Press, 2018).

[6] Andersen, L. W. *et al.* Population structure and gene flow of the Atlantic walrus (*Odobenus rosmarus rosmarus*) in the eastern Atlantic Arctic based on mitochondrial DNA and microsatellite variation. *Mol. Ecol.* **7**, 1323–1336 (1998). 9787444 10.1046/j.1365-294x.1998.00455.x

[7] Anonymous. Fredning av hvalross. (Protection of walruses). Available from: Fiskeridepartementet, Øvre Slottsgate 2, P. O. B. 8188 Dep., 0032 Oslo, Norway (1952).

[8] Lydersen, C., Aars, J. & Kovacs, K. M. Estimating the number of walruses in svalbard from aerial surveys and behavioural data from satellite telemetry. *Arctic* **61**, 119–128 (2008).

[9] Norwegian Polar Institute. Walrus population in Svalbard. Environmental monitoring of Svalbard and Jan Mayen (MOSJ). https://mosj.no/en/indikator/fauna/marine-fauna/walrus/ (2022).

[10] Gjertz, I. & Wiig, Ø. Past and present distribution of walruses in Svalbard. *Arctic* **47**, 34–42 (1994).

[11] Kovacs, K., Aars, J. & Lydersen, C. Walruses recovering after 60+ years of protection in Svalbard, Norway. *Polar Res.* **33** (2014).

[12] Born, E. W. Reproduction in female Atlantic walruses (*Odobenus rosmarus rosmarus*) from north-west Greenland. *J. Zool.* **255**, 165–174 (2001).

[13] Sjare, B. L. & Stirling, I. The breeding behavior of Atlantic walruses, *Odobenus rosmarus rosmarus*, in the Canadian High Arctic. *Can. J. Zool.* **74**, 897–911 (1996).

[14] Fay, F. H., Ray, G. C. & Kibal’chich, A. A. Time and location of mating and associated behavior of the Pacific walrus, *Odobenus rosmarus divergens* Illiger. *NOAA Tech. Report* **12**, 89–99 (1984).

[15] Lowther, A. D., Kovacs, K. M., Griffiths, D. & Lydersen, C. Identification of motivational state in adult male Atlantic walruses inferred from changes in movement and diving behavior. *Mar. Mamm. Sci.* **31**, 1291–1313 (2015).

[16] Born, E. W. & Knutsen, L. O. Satellite-linked radio tracking of Atlantic walruses (*Odobenus rosmarus rosmarus*) in northeastern Greenland, 1989–1991. *Zeitschrift für Säugetierkunde* **57**, 275–287 (1992).

[17] Born, E. W., Aquarone, M., Knutsen, L. Ø. & Pedersen, L. T. Homing Behaviour in an Atlantic Walrus (*Odobenus rosmarus rosmarus*). *Aquat. Mamm.* **31**, 23–33 (2005).

[18] Lydersen, C., Lindgren, Å., Alfredsson, K. & Kovacs, K. M. A Walrus (*Odobenus rosmarus*) at the North Pole. *Aquat. Mamm.* **48**, 513–516 (2022).

[19] Freitas, C., Kovacs, K. M., Ims, R. A., Fedak, M. A. & Lydersen, C. Deep into the ice: over-wintering and habitat selection in male Atlantic walruses. *Mar. Ecol. Prog. Ser.* **375**, 247–261 (2009).

[20] Wiig, Ø., Gjertz, I. & Griffiths, D. Migration of walruses (*Odobenus rosmarus*) in the Svalbard and Franz Josef Land area. *J. Zool.* **238**, 769–784 (1996).

[21] Jay, C. V. & Hills, S. Movements of walruses radio-tagged in Bristol Bay, Alaska. *Arctic* **58**, 192–202 (2005).

[22] Acquarone, M. *et al.* Evaluation of etorphine reversed by diprenorphine for the immobilisation of free-ranging Atlantic walrus (*Odobenus rosmarus rosmarus* L.). *NAMMCO Sci. Pub.* **9**, 345–360 (2014).

[23] Griffiths, D., Wiig, Ø. & Gjertz, I. Immobolization of walrus with etorphine hydrochloride and zoletil®. *Mar. Mamm. Sci.* **9**, 250–257 (1993).

[24] Brunson, D. B. Walrus in *Zoo Animal and Wildlife Immobilization and Anesthesia* (ed. West, G., Heard, D. & Caulkett, N.) 673–678 (John Wiley & Sons, Inc., 2014).

[25] Gales, N. J. Chemical restraint and anesthesia of pinnipeds: A review. *Mar. Mamm. Sci.* **5**, 228–256 (1989).

[26] Ølberg, R. A., Kovacs, K. M., Bertelsen, M. F., Semenova, V. & Lydersen, C. Short duration immobilization of Atlantic Walrus (*Odobenus rosmarus rosmarus*) with etorphine, and reversal with naltrexone. *J. Zool. Wildl. Med.* **48**, 972–978 (2017). 10.1638/2016-0232R.1 29297843

[27] CES. Animal Care, Equipment and Services. https://animal-care.com/product/teledart-model-rd706-rifle/ (2024).

[28] R Core Team. R: A language and environment for statistical computing. R Foundation for Statistical Computing, Vienna, Austria. https://www.R-project.org/ (2022).

[29] Jonsen, I. D. *et al.* aniMotum, an R package for animal movement data: Rapid quality control, behavioural estimation and simulation. *Methods Ecol. Evol.* **14**, 806–816 (2023).

[30] Sumner, M. D., Wotherspoon, S. J. & Hindell, M. A. Bayesian estimation of animal movement from archival and satellite tags. *PLoS ONE* **4**, e7324 (2009). 19823684 10.1371/journal.pone.0007324 PMC2758548

[31] Calenge, C. The package “adehabitat” for the R software: A tool for the analysis of space and habitat use by animals. *Ecol. Model.* **197**, 516–519 (2006).

[32] Fieberg, J. & Kochanny, C. O. Quantifying horme-range overlap: the importance of the utilization distribution. *J. Wildl. Manag.* **69**, 1346–1359 (2005).

[33] Thieurmel, B. & Elmarhraoui, A. A: Compute Sun Position, Sunlight Phases, Moon Position and Lunar Phase. Package 'suncalc'. https://CRAN.R-project.org/package=suncalc (2022).

[34] MET. Ice Service charts. https://cryo.met.no/en/latest-ice-charts (2023).

[35] Ross, N. fasterize: Fast Polygon to Raster Conversion, version 1.0.5. https://CRAN.R-project.org/package=fasterize (2023).

[36] Hijmans, R. J. terra: Spatial Data Analysis, version 1.7–65. https://CRAN.R-project.org/package=terra (2023).

[37] Pinheiro, J., Bates, D. & Team, R. C. nlme: Linear and Nonlinear Mixed Effects Models. R package version 3.1–162. https://CRAN.R-project.org/package=nlme (2023).

[38] Jonsen, I. D. *et al.* Movement responses to environment: fast inference of variation among southern elephant seals with a mixed effects model. *Ecology* **100**, e02566 (2019). 30467837 10.1002/ecy.2566

[39] Falk-Petersen, S. *et al.* Physical and ecological processes in the marginal ice zone of the northern Barents Sea during the summer melt period. *J. Mar. Syst.* **27**, 131–159 (2000).

[40] Vinje, T. & Kvambekk, A. S. Barents Sea drift ice characteristics. *Polar Res.* **10**, 59–68 (1991).

[41] Kovacs, K. M., Lemons, P., MacCracken, J. G. & Lydersen, C. Walruses in a time of climate change. *Arctic Rep. Card* **2015**, 66–74 (2015).

[42] Wiig, Ø., Born, E. W., Gjertz, I., Lydersen, C. & Stewart, R. E. A. Historical sex-specific distribution of Atlantic walrus (*Odobenus rosmarus rosmarus*) in Svalbard assessed by mandible measurements. *Polar Biol.* **31**, 69–75 (2007).

[43] Udevitz, M. S., Taylor, R. L., Garlich-Miller, J. L., Quakenbush, L. T. & Snyder, J. A. Potential population-level effects of increased haulout-related mortality of Pacific walrus calves. *Polar Biol.* **36**, 291–298 (2013).

[44] Goertz, C. E. C., Polasek, L., Burek, K., Suydam, R. & Sformo, T. Demography and pathology of a pacific walrus (*Odobenus rosmarus divergens*) mass-mortality event at Icy Cape, Alaska, September 2009. *Polar Biol.* **40**, 989–996 (2017).

[45] Wassmann, P. *et al.* Food webs and carbon flux in the Barents Sea. *Prog. Ocean.* **71**, 232–287 (2006).

[46] Søreide, J. E. *et al.* Sympagic-pelagic-benthic coupling in Arctic and Atlantic waters around Svalbard revealed by stable isotopic and fatty acid tracers. *Mar. Biol. Res.* **9**, 831–850 (2013).

[47] Fossheim, M. *et al.* Recent warming leads to a rapid borealization of fish communities in the Arctic. *Nat. Clim. Change* **5**, 673–677 (2015).

[48] Abrahms, B. *et al.* Climate mediates the success of migration strategies in a marine predator. *Ecol. Lett.* **21**, 63–71 (2018). 29096419 10.1111/ele.12871

[49] Skoglund, E. G., Lydersen, C., Grahl-Nielsen, O., Haug, T. & Kovacs, K. M. Fatty acid composition of the blubber and dermis of adult male Atlantic walruses (*Odobenus rosmarus rosmarus*) in Svalbard, and their potential prey. *Mar. Biol. Res.* **6**, 239–250 (2010).

