# Publisher Correction: Interannual site fidelity by Svalbard walruses

**DOI:** 10.1038/s41598-024-78275-9

**Published:** 2024-11-12

**Authors:** Lonnie Mikkelsen, Kit M. Kovacs, Marie‑Anne Blanchet, Gary Brodin, Christian Lydersen

**Affiliations:** 1 Norwegian Polar Institute, Framsenteret, Hjalmar Johansens Gate 14, 9296 Tromsø, Norway; 2 Pathtrack Ltd, Unit 1, Chevin Mill, Leeds Road, Otley, LS21 1BT UK

Correction to: *Scientific Reports* 10.1038/s41598-024-66370-w, published online 09 July 2024

The Funding section in the original version of this Article was incorrect.

“Open access funding provided by Norwegian Polar Institute.”

now reads:

“The tag development and field activities involved in this project were funded by the Norwegian-Russian Environmental Commission. Analyses and manuscript drafting were undertaken with financial support from The Norwegian Research Council project “ARK” (nfr # 313678) and the EU FACE-IT (The Future of Arctic Coastal Ecosystems – Identifying Transitions in Fjord Systems and Adjacent Coastal Areas) project. FACE-IT has received funding from the European Union’s Horizon 2020 research and innovation programme under grant agreement No 869154. Additionally staffing and logistical support were provided by the Norwegian Polar Institute.”

The original Article has been corrected.

