# Supplementary material for: Interannual site fidelity by Svalbard walruses
Source: Sci Rep. 2024 Jul 9;14:15822. doi: 10.1038/s41598-024-66370-w (PMC11233647; doi:10.1038/s41598-024-66370-w)

Figure S1. Distribution of depths in core areas (50 % utilisation distribution) of Svalbard male walruses. *a*) summer and *b*) winter.


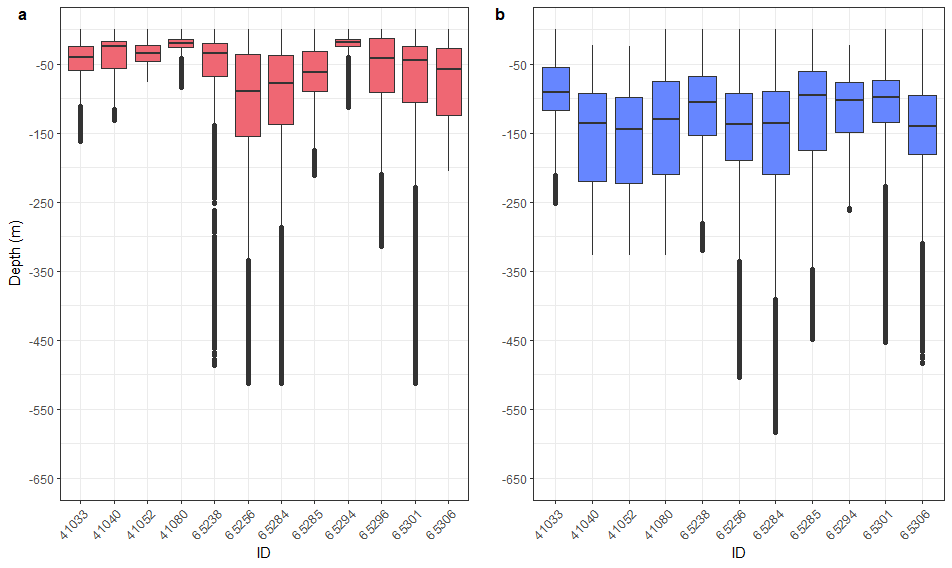


Figure S2. Average daily sea ice concentration of Svalbard walruses over 14 days before migration. Ice concentration is based on positions of the walruses before migration to the winter areas, separated by the tagging location at either Lågøya (North-Svalbard) or Sletteøya (South-Svalbard). The bottom graph shows average daily ice concentration prior to migration from winter areas to summer areas for all walruses. Data from all years are pooled into the graphs.


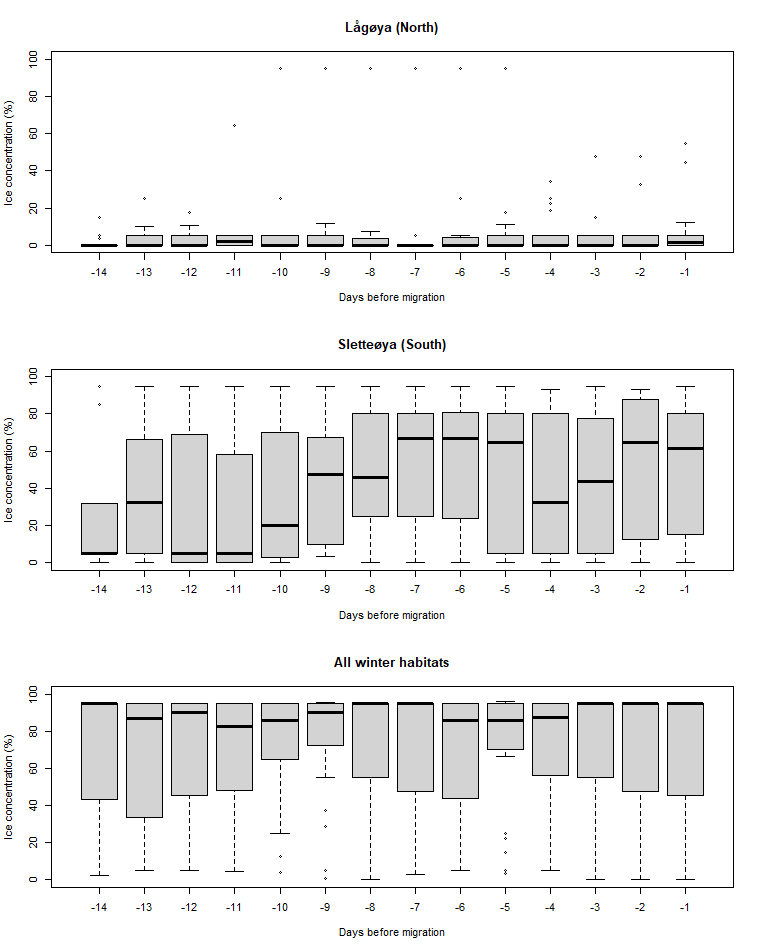

Supplement: Supplementary file 1 — Supplementary Figures. [file 41598_2024_66370_MOESM1_ESM.docx]
